# A New Proposal for the Pathogenic Mechanism of Non-Coeliac/Non-Allergic Gluten/Wheat Sensitivity: Piecing Together the Puzzle of Recent Scientific Evidence

**DOI:** 10.3390/nu9111203

**Published:** 2017-11-02

**Authors:** Valentina Leccioli, Mara Oliveri, Marcello Romeo, Massimiliano Berretta, Paola Rossi

**Affiliations:** 1 Department of Biology and Biotechnology “L. Spallanzani”, University of Pavia, via Ferrata 1, 27100 Pavia, Italy; mara.oliveri@libero.it; 2 C.E.R.H.M. Center for Experimental Research for Human Microbiome Ludes H.E.I., Pietro Stiges Palace, Strait Street, 1436 Valletta, Malta; drmarcelloromeo@gmail.com; 3 Department of Medical Oncology, CRO-Aviano, National Cancer Institute, Via Franco Gallini 2, 33081 Aviano, Italy; mberretta@cro.it

**Keywords:** non-coeliac gluten/wheat sensitivity, pathogenic mechanism, butyrate, amylase trypsin inhibitors, lipopolysaccharide, intestinal alkaline phosphatase, microbiota

## Abstract

Non-coeliac/non-allergic gluten/wheat sensitivity (NCG/WS) is a gluten-related disorder, the pathogenesis of which remains unclear. Recently, the involvement of an increased intestinal permeability has been recognized in the onset of this clinical condition. However, mechanisms through which it takes place are still unclear. In this review, we attempt to uncover these mechanisms by providing, for the first time, an integrated vision of recent scientific literature, resulting in a new hypothesis about the pathogenic mechanisms involved in NCG/WS. According to this, the root cause of NCG/WS is a particular dysbiotic profile characterized by decreased butyrate-producing-*Firmicutes* and/or *Bifidobacteria*, leading to low levels of intestinal butyrate. Beyond a critical threshold of the latter, a chain reaction of events and vicious circles occurs, involving other protagonists such as microbial lipopolysaccharide (LPS), intestinal alkaline phosphatase (IAP) and wheat α-amylase trypsin inhibitors (ATIs). NCG/WS is likely to be a multi-factor-onset disorder, probably transient and preventable, related to quality and balance of the diet, and not to the presence of gluten in itself. If future studies confirm our proposal, this would have important implications both for the definition of the disease, as well as for the prevention and therapeutic-nutritional management of individuals with NCG/WS.

## 1. Introduction

Non-coeliac/non-allergic gluten/wheat sensitivity (NCG/WS) is a clinical condition described for the first time in 1978 by Ellis and Linaker [1], and then in 1980 by Cooper et al. [2], who reported cases of patients presenting gluten-responsive clinical picture in absence of coeliac disease (CD). However, it was only in 2012 that NCG/WS has been considered to be within the gluten-related disorder (GRD) spectrum, together with CD and wheat allergy (WA) [3]. In 2012, a consensus on new nomenclature and classification of gluten-related disorders has been published [3], after the first of three International Expert Meetings on GRD, all leading to related publications [3,4,5] that outlined NCG/WS main clinical and diagnostic features. While CD is a chronic small intestinal, autoimmune enteropathy triggered by gluten and related prolamines in genetically predisposed individuals, and WA is an adverse immunologic reaction to wheat proteins, NCG/WS is “a condition in which symptoms are triggered by gluten ingestion, in the absence of celiac-specific antibodies and of classical celiac villous atrophy, with variable Human Leukocyte Antigen (HLA) status and variable presence of first generation anti-gliadin antibodies (AGA)” [3,4]. HLA-DQ2 and -DQ8 are the genetic markers most strongly associated with CD, being positive in approximately 95% of coeliac patients. HLA haplotypes are found positive in about 50% of NCG/WS patients, only slightly higher than 30% of the general population [4].

The recent renewed interest of scientific community in NCG/WS is due to an increasing number of patients, not affected by CD or WA, referring intestinal and extra-intestinal symptoms after gluten/wheat ingestion; despite the initial skepticism on its very existence as a discrete entity, NCG/WS has been recognized as an independent disorder of clinical, social, and economic relevance. However, there are still questions about its separation from CD [3,4,5,6,7,8,9]. This immune-mediated disorder [10] affects individuals for whom CD and WA have been ruled out according to the respective current diagnostic criteria [5,11]. It is characterized by heterogeneous and not specific gastrointestinal (GI) symptoms, including abdominal pain, bloating, bowel habit abnormalities (diarrhea, alternating bowel and constipation), and extra-intestinal symptoms, including chronic tiredness, headache, ‘foggy mind’, joint and muscle pain, limb numbness, eczema or skin rash, depression, anemia, of variable severity, occurring within hours or a few days after the ingestion of gluten-containing foods; symptoms improve or rapidly disappear with the exclusion of the latter and recur following their reintroduction [3,4,5,11].

In the last few years, several studies have suggested that both innate and adaptive immunity are involved [9,10,12], but there still remains an absence of confirmed and validated specific biomarkers. Therefore, according to the Salerno Experts, a diagnosis of NCG/WS should be made after a positive double-blind placebo-controlled gluten challenge with crossover (DBPCC) [5,11]. However, this procedure has been shown to be an “imperfect gold standard” [13,14], and in daily clinical practice, the diagnosis remains based on the evaluation of symptoms, the exclusion of CD and WA and improvement in symptoms after elimination of gluten/wheat from the diet (the latter of which is often influenced by placebo effects) [9,15]. Diagnostic difficulty, the lack of correct and scientific diagnostic work-up by some clinicians, and the considerable attention have given by the media to this “young” disorder, have contributed to the spread of the “self-diagnostic” phenomenon and the devotion of many people to gluten-free diets (GDF), which are often self-administered [4,9,12,16,17]. In fact, as for CD and WA, a GFD is the only possible treatment for NCG/WS, to date, but the severity and duration of this diet are not yet well defined, because of the uncertainty about the pathogenesis and triggers, the lack of specific biomarkers, and the strong inter-individual differences among patients [11]. All these factors also hamper the performance of accurate research because of the resulting heterogenous criteria for the selection of patients for the studies, and the following poorly comparable data [12]. Although risk factors for this disorder have not yet been identified, NCG/WS seems to be more common in females and in young/middle age adults [12]. Because of the “self-diagnostic” phenomenon and the absence of biomarkers, the overall prevalence of NCG/WS remains vague and possibly ranging from 0.6% to 6% [4,9]; the prevalence in children is still unknown [12].

There are increasing doubts among the scientific community regarding whether gluten is the trigger of NCG/WS; these doubts are supported by several studies [13,18,19,20,21,22,23,24]. It has been suggested that other molecules could determine the onset of NCG/WS [25]; possible candidates are fermentable oligo-, di-, monosaccharides and polyols (FODMAPs) [18], wheat amylase trypsin inhibitors (ATIs) [19,20,21], wheat germ agglutinin (WGA) [26,27] and exorphins [28].

Regarding the pathogenic mechanism of NCG/WS, a recent study conducted by Uhde et al. [29] clarified some aspects of this condition [10]. This study also suggested that some biomarkers (fatty acid-binding protein 2 or FABP2, soluble CD14 or sCD14, lipopolysaccharide (LPS)-binding protein or LBP, endotoxin-core antibodies or EndoCAb IgM, anti-flagellin IgM and IgG) could be useful as possible diagnostic tools, although they are yet to be confirmed and validated. Uhde demonstrated the presence of enterocyte injury and translocation of microbial components from the intestinal lumen to the blood circulation, resulting in activation of the systemic innate and adaptive immune response [29]. This study confirmed the already verified [30,31] existence of an increased intestinal permeability in individuals with NCG/WS, and provided evidence for a possible pathogenic role of the intestinal microbiota [12]. One of the main questions that remains to be answered is what induces the increased intestinal permeability, allowing microbial and food-borne antigens to cross into the lamina propria, and how does this occur [12].

Starting from the best of our current knowledge, in the present paper we connect some recent scientific evidence, which were never been expressly linked together till now, and propose a new hypothesis on the pathogenic mechanism of NCG/WS, by reconstructing the possible “chain reaction” involved in the onset of this disease. Finally, we provide some starting points for further research that, if confirmed by future studies, could imply important changes both to the substantial definition of the disease and to the therapeutic-nutritional management of individuals with NCG/WS.

## 2. Scientific Background

### 2.1. Diagnostic Difficulties

Because a clear pathogenic mechanism and specific, confirmed and validated biomarkers are yet to be identified, the evaluation of symptoms remains fundamental for diagnosis of NCG/WS [9,10,12]. Unfortunately, the symptoms are also shared by CD [3,14], irritable bowel syndrome (IBS) [4,5,14] and non-immunoglobulin E (IgE)-mediated food allergies [4,14,32,33,34], and, as for CD, symptoms must be considered a poor indicator and predictor of the disease [29,35,36].

The current diagnostic criteria for NCG/WS [5,11] are insufficient for a certain identification of sensitive individuals, both for clinical practice [9,13] and, in our opinion, for research. They are based on the exclusion of CD and WA, and on the clinical responsiveness of individuals to a GFD and gluten rechallenge [5,11]. However, CD and WA cannot always be adequately excluded [14]. According to a recent systematic review [8], up to 20% of NCG/WS patients in literature were eventually reclassified as coeliac patients, after re-evaluation after gluten challenge, or advanced diagnostic investigations, such as characterization of γδ intraepithelial lymphocytes (IELs), immunohistological detection of anti-tTG2 IgA, duodenal aspirate or biopsy culture, and HLA-DQ2-gliadin tetramer test [9,13]. This subgroup of NCG/WS patients was characterized by lymphocytic enteritis (LE, representing Marsh 1 lesion level), negative serology for CD (anti-endomysial-EmA- and anti-tissue transglutaminase 2-tTG2-IgA), and positive genetics for CD (HLA-DQ2/DQ8 haplotype) [9,13]. Furthermore, according to the current diagnostic criteria, non-IgE-mediated WA can fall within the NCG/WS spectrum [4,32,33,34]. Non-IgE-mediated food allergies have different systemic and GI symptoms, similar to NCG/WS in terms of quality and time of onset. Without any biomarker, aside from an increased number of eosinophils in normal intestinal mucosa, they can be diagnosed by a positive response to an elimination diet followed by a DBPCC [14,32,34].

With regard to the DBPCC proposed to confirm the diagnosis of NCG/WS [5], besides being cumbersome, time consuming and costly, it is subject to important precebo, placebo, and nocebo effects and presents with a series of parameters that still need standardization and validation [9,13,14]; moreover, Molina-Infante and Carroccio show that more than 80% of recruited patients undergoing a DBPCC cannot reach a formal diagnosis of NCG/WS, 40% have a nocebo response and only 16% show gluten-specific symptoms, reaching 30% when the challenge is performed with wheat [13]. These findings highlight why DBPCC remains an “imperfect gold standard” for NCG/WS, and raise doubts about the role of gluten in the actual triggering of the disease, as suggested by many other studies [13,18,19,20,21,22,23,24].

The shared intestinal manifestations also make it hard to distinguish NCG/WS from IBS [14], a chronic functional GI disorder diagnosed exclusively on the basis of non-specific clinical characteristics [37,38,39]. Many researchers suggest that NCG/WS may be a subgroup of IBS, rather than an independent clinical entity. This hypothesis is supported by the fact that the clinical picture in NCG/WS is almost always dominated by some GI symptoms, among those previously mentioned, most of which are also present in IBS, and that FODMAPs, rather than gluten, are responsible for them [6,18,25,39,40,41,42,43]. FODMAPs are present in grains and related products (especially those gluten-related), legumes, fruit, vegetables, milk and honey [9,44,45], and could in fact induce distension of the intestine because they are osmotically active molecules and fermentative substrates [25,40,46]. However, FODMAPs are known to inhibit rather than cause inflammation, by inducing beneficial changes in the intestinal microbiota and generation of short-chain fatty acids (SCFAs) [42,46,47,48]. It is unlikely that FODMAPs are the sole responsible for symptoms reported by NCG/WS subjects [42,49]: many individuals in clinical remission with a GFD do continue to ingest FODMAPs from legumes [18], containing quantities of these carbohydrates that are comparable to those of gluten-containing grains [44,45]. In our opinion, rather than triggers, FODMAPs should be considered as possible additional elements of disturbance that, in this specific case, could exacerbate symptoms associated with the gut lumen which is already compromised, due to other causes. Moreover, it is possible that individuals with NCG/WS could also have, at the same time, a lack of one or more enzymes for the digestion of FODMAPs or other nutrients; this would also explain cases of people defining themselves NCG/WS because of an association between the appearance of typical manifestations and the ingestion of gluten-containing foods, but then continuing to report persistent symptoms despite adhesion to a GFD [41]. In this regard, Balakireva [50] claims that both conditions could coexist independently, without necessarily sharing a common pathophysiological basis.

### 2.2. ATIs as a Trigger of NCG/WS?

Recently, the scientific community has focused on the possible role of ATIs in GRDs [19,20,21,51,52,53]. Wheat α-amylase trypsin inhibitors belong to the family of water soluble albumins [21,53], which, together with globulins, represent 10–20% of total wheat proteins [19]; in the endosperm of plant seeds, they support the natural defense against parasites and insects and may regulate starch metabolism during seed development and germination. They are a family of compact protease-resistant proteins with strong disulfide bonds and high secondary structural homology, that copurify specifically with ω-gliadins. ATIs can be grouped into three subfamilies of approximately 50–60, 24–30 and 12–15 kDa [19,21].

Studies on immune stimulating activity of ATIs have been conducted in human and murine macrophages, monocytes and dendritic cells (DCs), in cultures of coeliac intestinal biopsies as well as in vivo in mice [19,21]. These studies have demonstrated that ATIs (in particular CM3 and 0.19 types, of about 15 KDa) are strong activators of dendritic cells (DCs), macrophages and monocytes [19,21]. On these cells they engage the TLR4-MD2-CD14 complex, thereby activating both the classical (nuclear factor kappa B or NF-kB) and the non-classical (interferon responsive factor 3 or IRF-3) pathway. It results in an up-regulation of maturation markers and the early release of innate proinflammatory cytokines, IL-1β, IL-6, TNFα, and then later, IL-8 and MCP-1 [19,21]. Contrary to wild type mice, studies have shown that TLR4- or TLR4-signalling-deficient mice do not show intestinal and systemic innate immune activation after oral challenge with ATIs [20,21]. Furthermore, the in vitro and in vivo experiments performed by Junker [21] found that gliadins or gliadin peptides, such as p31–43 or p31–49, are not innate immunity stimulators. Zevallos [19] reported that in mice fed with an ATI/gluten-free diet for four weeks, a single gavage of about 12 mg/mouse of commercial gluten (containing 0.2 mg ATIs) increased parameters of innate inflammation along the whole intestine; this did not happen in mice challenged with the same dose of gluten that was 70% de-enriched of ATIs by prior extraction. This appears to indicate that pure dietary gluten itself has no relevant immune stimulating activity in normal mice, in contrast to what has been suggested in ex vivo settings [19].

Intestinal myeloid cells probably sense ATIs through DCs body extensions into the gut lumen while probing it for the presence of antigens and/or through an active transport of intact ATIs across the intestinal epithelial layer, as occurs with gliadin peptides [20,54,55]. A direct interaction between ATIs and TLRs may also occur on the surface of enterocyte membrane, leading to the development of intestinal inflammation [56].

Schuppan [20] suggests that ATIs could have an adaptive adjuvant effect on pre-existing intestinal inflammation, in addition to promoting an innate immune response. A healthy adult person with a daily consumption of 150–250 g of wheat flour, therefore exposed to about 0.5–1 g of ATIs, would have a modest or moderate innate intestinal immune activation without development of symptoms, thanks to immune tolerance mechanisms [20,21]. According to Schuppan [20], individuals with NCG/WS could be those with pre-existing or chronic inflammatory diseases in whom the sensing/uptake of ATIs is increased, probably as a consequence of the disruption of intestinal homeostasis and barrier integrity. Thus, even modest activation of innate immunity could exacerbate inflammatory conditions already present, by indirectly promoting an adaptive response through the strengthening of pre-existing antigenic exposition of antigen presenting cells (APC) to T cells. According to Schuppan, such adaptive responses would occur in the gut, as well as in nearby or more remote lymph nodes or lymphatic organs, resulting in the typical NCG/WS extra-intestinal inflammation [20]. Zevallos [19] observed such adjuvant effect of nutritional ATIs in mice with pre-existing dextran sodium sulfate (DSS)-induced small intestinal or colonic inflammation. However, as suggested by Zevallos [19], further preclinical and clinical studies in human are warranted to assess the effect of an ATIs-free diet on intestinal and extra-intestinal inflammatory diseases.

In light of these findings, the recently proposed “FODMAPs hypothesis” concerning NCG/WS would be hardly sustainable, as discussed by Zevallos [19]. As previously mentioned, FODMAPs cannot induce the inflammatory responses occurring in NCG/WS patients, but rather could contribute to “mechanically” worsen their symptoms. In general, scientific evidence seems to better support the role of ATIs as a trigger of NCG/WS, which seems likely to be an immune-mediated disorder [10].

### 2.3. Microbial Lipopolysaccharide, Intestinal Alkaline Phosphatase and Intestinal Permeability

TLR4 activation by ATIs resembles that induced by LPS, the strongest TLR4 agonist. In fact, TLR4 activation by ATIs is CD14-dependent and engages MyD88-mediated downstream signaling (leading to activation of NF-kB and transcriptional upregulation of proinflammatory cytokines and chemokines such as IL-8, TNFα and MCP-1), or TRIF-mediated signaling (resulting in secretion of type I interferons and RANTES) [21].

LPS is the major cell wall component of Gram negative bacteria, consisting of a hydrophobic portion (lipid A) and a hydrophilic portion (an oligosaccharidic core plus an antigenic polysaccharide called O-antigen); it is released from bacterial cell wall by shedding or bacterial lysis [57]. As opposed to ATIs, ingested and luminal LPS is usually completely inactivated by gastric acids and intestinal alkaline phosphatase (IAP) [19,20,21].

The latter is an important brush-border enzyme involved in preventing intestinal inflammation and preserving the gut microbiota homeostasis. Mainly produced by the proximal small intestine epithelial cells, it is secreted both luminally and basolaterally and inactivates, by dephosphorylation, the microbial components normally present at high concentrations in the gut lumen [58,59,60,61]. In particular, IAP may play a pivotal role in the maintenance of intestinal barrier integrity by detoxification of LPS [62]. IAP is downregulated in settings where gut barrier dysfunctions are critical in the development of diseases, such as inflammatory bowel disease (IBD): low levels of IAP have been found in inflamed colonic biopsies of patients with Crohn’s disease and ulcerative colitis (UC) [63,64]. Expression levels and activity of duodenal IAP were found to be particularly low, and related to the disrupted intestinal barrier integrity, in severe cases of CD in young patients [62,65]. IAP knock-out (KO) mice show higher LPS influx to the systemic circulation [66]. Exogenous IAP supplementation prevents the development of colitis in both human and mice [67,68], and prevents LPS-induced barrier dysfunctions in vitro [58].

In this regard, in healthy individuals with intact intestinal barriers, plasma concentrations of LPS range from undetectable levels up to 0.2 ng/mL; a variety of physiological factors can result in permeability alterations that lead to plasma LPS levels ranging about 1–2 ng/mL, while patients with intestinal permeability disorders such as necrotizing enterocolitis (NEC) and IBD can reach levels between 2 and 10 ng/mL [69,70,71,72,73,74,75,76,77,78]. Guo et al. [57] found that relatively low but physiologically and clinically relevant levels of LPS in the interstitial fluid of enterocytes can lead to a reversible, time-dependent increase in paracellular permeability in vitro (filter-grown Caco-2 monolayers) and in an in vivo (mouse intestinal perfusion) intestinal epithelial model system, without inducing cell death [57]. This occurs through a TLR4-MD2-CD14-mediated intracellular mechanism, engaged by LPS linked to LBP, and involves a TLR4-dependent up-regulation of CD14 membrane expression [57]. A variety of physiological factors such as prolonged physical exertion, high-fat diet, physiological stresses, or intestinal permeability disorders can readily achieve these concentrations of LPS in the interstitial fluid of enterocytes [57]. The consequent paracellular permeability variation occurs within four or five days [57], and can be dynamically regulated by altering both the expression levels and localization of tight junction proteins (TJPs) [58]. Inflammatory pathways, such as LPS-induced NF-kB activation and the consequent production of cytokines, result in disrupted levels and localization of TJPs [79,80]. This, in turn, can increase the passage of intestinal contents to the gut mucosa and to the systemic circulation [57,58]. As circulating LPS is an important determinant of the inflammatory response and multi organ failure, it could therefore play an important role in further deterioration and prolongation of intestinal TJ barrier defects in intestinal permeability disorders and inflammatory gut diseases [57].

Regarding intestinal permeability, the early belief that it was reduced in NCG/WS [81] has been definitively rejected [9]. Several studies have shown that an increased one is present even in non-coeliac patients, in particular in NCG/WS [30,31], IBS [31,82], and generic “non-coeliac” patients with persistent dyspeptic complaints [83]. Barbaro [31] proposed that zonulin could play a role in the pathophysiology of NCG/WS because of increased zonulin serum levels and a correlation with symptoms found in NCG/WS patients, suggesting disassembly of TJs. Hollon [30] analyzed intestinal permability of duodenal biopsy explants in four study groups (coeliacs with active disease (ACD), coeliacs in remission, non-coeliac gluten sensitives and non-coeliac dyspeptic controls) through the measurement of transepithelial electrical resistance (TEER). This study found an increase in permeability in all groups after pepsin-trypsin digested gliadin (PT-gliadin) exposure, compared to the media alone, with a greater increase in individuals with ACD and with NCG/WS. Finally, the findings by Uhde [29] about damage to enterocytes and translocation of microbial components from the lumen to the intestinal mucosa and blood circulation, further support the presence of increased gut permeability in NCG/WS. Uhde [29] also found an association between improvement in symptoms after GFD and normalization of biomarkers levels, although the magnitude of the latter did not correlate significantly with that of the symptoms scores. The damage to enterocytes was deduced by serological levels of FABP2 [29], a cytosolic protein specific to intestinal epithelial cells, rapidly released into systemic circulation after cellular damage, reflecting changes in the rate of enterocyte turnover [84,85,86,87]. In individuals with NCG/WS, elevated levels of circulating FABP2 are comparable to those of individuals with CD, and correlate with systemic innate and adaptive immune responses to microbial antigens. These are respectively evidenced by the significant increase in serological levels of soluble CD14 (sCD14) and LBP, and of antibodies towards LPS (EndoCAb IgM) and flagellin (anti-flagellin IgM and IgG). All these serum levels were significantly increased in NCG/WS patients, compared both to healthy and CD cohorts, except anti-flagellin IgM and IgG which were significantly increased only when compared to healthy controls [29]. It has been reported that LBP and sCD14 bind to circulating LPS and, depending on their relative concentrations, transfer LPS either to the TLR4-MD2-CD14 complex on myeloid cells, resulting in inflammatory stimulation, or to high density lipoproteins (HDL), lowering bioactivity of LPS [88,89]. Plasma lipoproteins, and in particular HDL, play an important role in neutralization of circulating LPS by transporting it to the liver for metabolization and excretion in the bile. In humans, low HDL plasma levels were found in septic patients, while raised ones are associated with a reduction in LPS-induced inflammation [90]. Low HDL levels could favour the transfer of LPS to the TLR4-MD2-CD14 complex, favouring inflammatory processes; by failing or decreasing the passage of LPS to HDL, peripheral inflammatory events would have a “green light”. In this regard, it would be interesting to assess HDL levels in individuals with NCG/WS to demonstrate a correlation between presence and intensity of symptoms and their improvement after dyslipidemia correction.

We must highlight that in the mentioned studies on intestinal permeability in NCG/WS patients, issues about different inclusion/exclusion criteria recur, as in most of the studies about this condition, due to the lack of specific confirmed biomarkers.

## 3. New Hypothesis on the Pathogenic Mechanism of NCG/WS

The intestinal epithelial surface is in constant contact with the enteroma; in diseases such as IBD, NEC and HIV infections there is a correlation between compromised epithelial integrity and immune responses consequent to translocation of microbial components from the intestinal lumen to the blood circulation [88,91]. Human microbiota, mainly composed of species belonging to *Firmicutes* and *Bacteroidetes* phyla, is suggested to play immunological, structural and metabolic functions such as, for example, the preservation of GI barrier integrity through the production of SCFAs [88].

Among these, butyrate is the major source of energy for the colonic mucosa, in turn promoting epithelial cell differentiation and injury repair; it also seems to play an important protective role in colorectal carcinogenesis. Butyrate stimulates the secretion of mucin and epithelial antimicrobial peptides, the synthesis of TJPs, and prevents microbial translocation [92,93,94,95]. Recently Yan and Ajuwon [92] found that butyrate significantly and dose-dependently protects intestinal barrier integrity from LPS-induced impairment. This effect was indicated by the restoration of paracellular permeability, measured by TEER and paracellular uptake of fluorescein isothiocyanate-dextran (FITC-dextran), and was carried out through the selective stimulation of TJPs and downregulation of TLR-4 expression.

Butyrate-producing bacteria are a functional group within the human gut microbial population [96]. Most of these bacteria belong to *Firmicutes* phylum, in particular Clostridial Clusters IV and XIVa (saccharolytic, strictly anaerobic Gram positive bacteria). Numerically, two of the most important groups appear to be *Faecalibacterium prausnitzii* (Clostridial cluster IV), and *Eubacterium rectale*/*Roseburia* spp. (Clostridial cluster XIVa) with a detection rate in faeces of healthy adults of about 2–15% compared to total bacteria [96,97]. Human studies are often limited to faecal samples analysis that, however, do not provide information about microbiota in the mucus layer, where butyrate-producing *Firmicutes* (b-p*F*) mainly reside, according to a validated in vitro gut model (M-SHIME) [98]. In fact, Clostridial cluster XIVa and IV species constitute respectively 59% and 19% of the mucin-adhered *Firmicutes* microbiota (94% of the mucin layer total community), with major representatives in *Roseburia intestinalis* and *Eubacterium rectale*. In contrast, *Proteobacteria* and *Bacteroidetes* prefer the lumenal milieu [98].

*Bifidobacteria* (Gram positive, anaerobic, saccharolytic bacteria belonging to the phylum *Actinobacteria*) contribute to the maintainance of adequate levels of intestinal butyrate by providing acetate and lactate to b-p*F*, in turn converting them into butyrate (cross-feeding interaction) [96,97,99].

Similarly, even mucins may indirectly serve as a growth substrate for b-p*F*, possibly via cross-feeding with mucin-degrading microbes, such as *A. muciniphila*, which provide partial breakdown products, acetate and lactate [98,100].

Low levels of b-p*F* are found in patients with IBD and their inflamed tissues compared to healthy individuals [96,101]. Low levels of b-p*F* and *Bifidobacteria* are also found in IBS and are associated with increased IBS symptoms [102,103]. Furthermore, the absence of butyrate in colonic tissue is associated with mucosal atrophy and coloncyte apoptosis [104,105].

In light of the above findings and considerations, a particular dysbiotic profile characterized by low levels of b-p*F* and *Bifidobacteria* could not provide an adequate butyrate level for enterocytes, resulting in reduced or absent butyrate trophic and protective effects. This would “starve” enterocytes, would negatively influence TJPs expression and localization, and would also predispose GI epithelial cells to possible cell damage or premature death. In fact, Uhde observed high FAPB2 serum levels in NCG/WS patients [29]. Moreover, as butyrate stimulates mucine secretion [95], a decrease or lack of butyrate could result in mucus layer alterations. Mucus is produced by goblet cells and forms a protective physical barrier covering enterocytes, thus preventing microrganisms and noxious substances from reaching epithelial surface [106]; so mucus layer impairment could further enhance the direct contact between enterocytes and microbial and food-borne antigens, and would create unfavorable conditions for b-p*F*, thereby creating a vicious circle. For example, recent in vivo and in vitro studies have revealed that IBD, in which the mucus layer becomes thinner and more discontinuous, is associated with low levels of mucosal butyrate producers, such as *Roseburia* and *Faecalibacterium*, indicating that a damaged mucus layer may lower the ecological fitness of specific butyrate producers [98].

Such a situation could allow sufficient LPS quantities to arrive in the interstitial fluid, resulting in a further increase in permeability, as explained by Guo [57]. In our opinion, this could constitute the pre-existing condition allowing the onset of NCG/WS. As a consequence, simultaneous paracellular translocation of microbial components and food-borne antigens, such as intact ATIs, could occur. Active ATIs would directly, and in greater quantities, stimulate the maturation of DCs, monocytes and macrophages of the GI tract, leading to activation of the innate immune response in the lamina propria [19,20,21]. Here, the stimulation of the TLR4-MD2-CD14 complex by ATIs would be additional to that of translocated LPS, amplifying the proinflammatory effect. Further, direct stimulation of the TLR4-MD2-CD14 complex on the surface of epithelial cells may occur to a greater extent because of the impaired mucus layer. Such a mechanism may also be amplified because TLR4 and CD14 expression and membrane co-localization are increased after LPS exposure in vitro and in vivo [57]. This would explain the typical local and rapid-onset intestinal symptoms of NCG/WS after the ingestion of gluten/ATI-containing foods. Uhde found that these translocated molecules could also arrive in the bloodstream and activate both innate and adaptive systemic immunity, as shown by the respective detection of serum LBP and sCD14, and EndoCAb and anti-flagellin antibodies. Translocated circulating antigens could bind to TLRs on other cells to trigger inflammatory responses in other parts of the body, explaining the extra-intestinal manifestations of NCG/WS [29].

Luminal LPS is usually completely inactivated by IAP: the latter, although exclusively secreted by brush border epithelial cells of the proximal small intestine, preserves its activity along the entire GI tract [107,108]. Several in vitro and in vivo studies in mice and rats have shown that IAP prevents adhesion of both pathogenic and commensal bacteria to the intestinal epithelial cells [109], prevents their translocation [107,110,111], inactivates possibly translocated pathogen-associated molecular patterns (PAMPs), and inhibits PAMP-induced NF-kB-mediated inflammatory responses [60,108]. IAP also stimulates gene expression of TJPs, such as ZO-1, ZO-2 and occludin, and their correct cellular localization, thus playing a direct role in intestinal barrier functionality [58]. However, we must highlight that butyrate is an inducer of IAP expression, and increases its activity [93,107,112,113]. Therefore, it is likely that a dysbiosis characterized by low levels of b-p*F* and/or *Bifidobacteria* could indirectly cause a decrease in IAP levels and activity, resulting in an insufficient detoxification of luminal microbial components, including LPS. The latter would have a greater green light for translocation across the previously described compromised NCG/WS GI barrier. In support of this hypothesis, also Goldberg [107] asserts that IAP silencing could result in impairment of the host’s ability to protect itself from luminal LPS exposure. Moreover, butyrate-induced IAP gene expression is inhibited by cytokines such as IL-1β and TNFα, and according to Malo [114], “cytokine-mediated IAP gene silencing may have important implications for gut epithelial function in the setting of intestinal inflammatory conditions”. Interestingly, IL-1β and TNFα are two of the cytokines produced after ATI stimulation [19], so that the GI inflammation, triggered by translocated antigens, could contribute to further silencing IAP, resulting in another vicious cycle.

In Junker’s in vivo experiments in mice to characterize the inflammatory activity of ATIs, orally ingested LPS was not found to cause increased transcription of cytokines, and thus, inflammation [21]. Junker, and later Schuppan and Zevallos, properly described this finding to be attributed to LPS inactivation by gastric acids and IAP activity [19,20,21]. In a paper on NCG/WS, Schuppan [20] defined ATIs as “the only relevant luminal TLR4 activator in the gastrointestinal tract”. In light of what discussed above, it could be that not only ATIs, but also luminal LPS from the resident microbiota, are involved in immune response activation in NCG/WS, thus accumulating their stimulating effects.

In NCG/WS individuals AGA are detected in a variable trend: AGA IgG are found in about half of patients [81,115], finding that created great discussion about their possible utility as a diagnostic tool for this disease [49,115,116,117,118], while AGA IgA are rarely detected [29,115]. Interestingly, in the study by Uhde [29] IgM responses to gliadin and microbial components (LPS and flagellin) were also investigated and found enhanced, in clear contrast with CD and healthy cohorts. Uhde suggests that acute microbial translocation from the gut would be expected to enhance the secretion of IgM antibodies in the periphery via a TLR9-dependent activation of B cells, independent of direct contact with respective antigens or T-cell involvement. IgM B cells would be further stimulated by direct interaction with specific antigens, such as translocated gliadins and microbial components, thus contributing to the observed IgM antibody responses [29]. However, according to our hypothesis, the triggers of NCG/WS are ATIs and microbial components, LPS in particular; we think that the host immune response would be mainly addressed against these ones, that should be further investigated in this regard. Even if our hypothesis is confirmed by future studies, further research will be necessary to understand pathophysiological and immunological events, and most of aspects of NCG/WS, such as variability in antibody reactivity among patients.

Contrary to CD patients, individuals with NCG/WS do not present villous atrophy or mucosal architecture abnormalities at duodenal level. For this reason, regarding NCG/WS, several authors have recently suggested possible elective damage sites aside from the duodenum [11,29,119]. Di Liberto et al. [119] suggested that in sensitive patients the immunological response could be greater in the colon rather than in the duodenal mucosa, because NCG/WS is generally characterized by GI symptoms also present in IBS, which is very often characterized by colonic mucosal inflammation [40,120,121]. In fact, Di Liberto observed innate lymphoid cell infiltrates (ILC1) in the rectal mucosa of individuals with NCG/WS, where a greater expression of proinflammatory cytokines such as IFNγ was also detected [119]. Uhde suggested the jejunum as an alternative damage location because it is the primary expression site of FABP2 [29]. On the basis of our proposal, we believe that the whole intestine could be involved in NCG/WS, maybe with an increasing trend in the colon. This hypothesis is based on several factors: first, the stimulating activity of ATIs progressively increases along the intestinal tract and is more marked in the colon rather than in the small intestine [19]; second, the colon contains the most “dense” and metabolically active microbiota within the adult GI tract, however the Clostridial cluster XIVa spp. is also present in the small intestine [97]. Regardless, it could be that epithelial changes associated with NCG/WS do not lead to overt remodelling of the mucosa, and therefore, they could require confocal laser endomicroscopy (CLE) for visualization [10,29].

**Figure 1:**
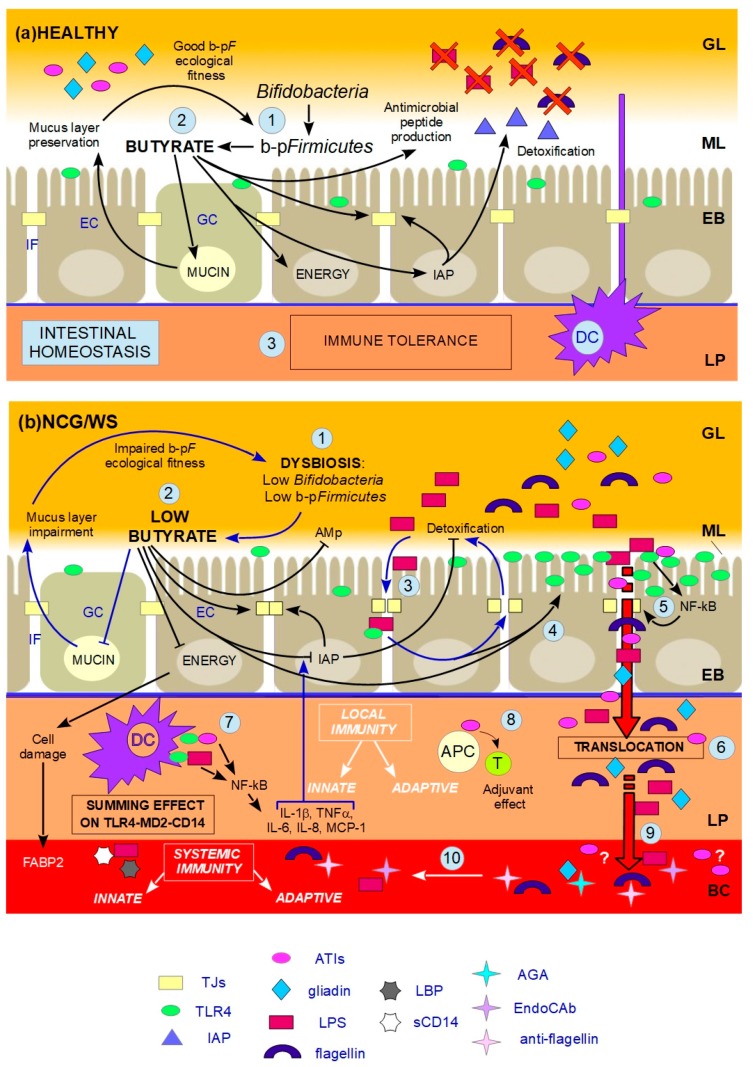
Schematic drawing that shows what happens in a healthy gut versus a non-coeliac gluten/wheat sensitivity gut according to our hypothesis. **NCG/WS** = non-coeliac gluten/wheat sensitivity; **EC** = enterocyte; **GC** = goblet cell; **IF** = interstitial fluid; **GL** = gut lumen; **ML** = mucus layer; **EB** = epithelial barrier; **LP** = lamina propria; **BC** = blood circulation; numbers in light blue balloons indicate the various steps in the chain reaction; → indicates stimulation; 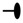 indicates inhibition. (**a**) HEALTHY: 1. Butyrate-producing *Firmicutes* (b-p*F*) provide adequate levels of butyrate in the ML and *Bifidobacteria* support the production of butyrate thanks to cross-feeding interactions with b-p*F*; 2. Butyrate in the ML, close to ECs, plays different trophic and protective functions: it stimulates GCs in the production of mucins, resulting in the preservation of the ML, and thus in a good b-p*F* ecological fitness. Butyrate constitutes the major energy supply for ECs; it favours the preservation of tight junctions (TJs) integrity by stimulating the expression and membrane co-localization of tight junction proteins (TJPs). Butyrate stimulates the production of antimicrobial peptides (AMp), and the expression and activity of intestinal alkaline phosphatase (IAP), thereby favouring the detoxification of microbial components; 3. All these functions together prevent that the content of the GL directly contacts and/or translocates across the EB, and, together with dendritic cells (DCs) which probe the GL for the presence of antigens, allow gut homeostasis and immune tolerance. (**b**) NCG/WS: (1) A dysbiosis characterized by low levels of b-p*F* and/or *Bifidobacteria* results in not sufficient levels of butyrate in the ML; (2) As a consequence, a chain reaction of events and vicious circles occur: the production of mucins is no longer stimulated, resulting in impairment of the ML. The consequent lowering of b-p*F* ecological fitness further promotes low levels of butyrate. ECs, without adequate energy source, run into inefficiency and cell damage, resulting in high serum levels of fatty acids binding protein 2 (FABP2). Moreover, TJs integrity is compromised, and the production of AMp is decreased. Low levels of butyrate also cause a decrease in the expression levels and activity of IAP; as a consequence, TJs integrity is further impaired, and the detoxification of microbial components is not sufficient; (3) The failed detoxification enables microbial lipopolysaccharide (LPS) to penetrate in the IF, where it increases paracellular permeability, with a consequent vicious cycle; (4) Furthermore, both LPS in the IF and low levels of butyrate upregulate toll-like receptors 4 (TLR4); (5) Because of the compromised ML, the lumenal content can reach EC surface. LPS and wheat amylase trypsin inhibitors (ATIs) can stimulate overexpressed TLR4, resulting in the production of NF-kB, and then later, inflammatory cytokines, which further damage TJs integrity; (6) Food-borne antigens and microbial components can cross the leaky EB; (7) In the LP, both translocated LPS and ATIs stimulate, at the same time, the TLR4-MD2-CD14 complex on myeloid cells, such as DCs, resulting in a local innate immune response with the production of inflammatory cyokines and chemokines. Among the latter, IL-1β and TNFα further inhibit the activity of IAP, thus maintaining this condition; (8) Moreover, ATIs have an adjuvant effect on possible pre-existing antigenic exposition of antigen-presenting cells (APC) to T-cells (T), triggering an adaptive immune response; (9) Microbial and food-borne antigens translocate in the BC (10), and trigger a systemic innate and adaptive immune response, respectively resulting in high serum levels of lipopolysaccharide-binding protein (LBP) and soluble CD14 (sCD14), and EndoCAb, anti-flagellin and anti-gliadin (AGA) antibodies.

In summary, the “culture medium” for the rooting of NCG/WS appears to be a pre-existing dysbiosis characterized by low levels of b-p*F* and/or *Bifidobacteria*, leading to a decrease in butyrate. Beyond a critical threshold of the latter, the gut would no longer be capable of dealing with different inflammatory stimuli, these being, an exogenous one from the diet (ATIs) and an endogenous one from the resident microbiota (LPS and other microbial components); as a consequence, it would begin a chain reaction as illustrated in Figure 1. In line with this, although no consensus has been agreed upon, Bennet [122] declared that in IBS patients there is a temporary decreased stability of the gut microbiota, leading to a dysbiosis generically defined as a weak tendency for a reduction in the beneficial bacteria of the gut countered with an increase in pathogenic species. Furthermore, Bennet highlighted the beneficial effects of butyrate and suggested that, although inconsistent, reduced levels of butyrate-producing *Eubacterium*, *Faecalibacterium* and *Roseburia* spp. could potentially be an ancillary cause of IBS symptoms in some patients [122]. Chassard [103] suggests that low levels of butyrate found in a subgroup of IBS patients could be due to the lowering of some butyrate-producing bacteria and may reduce the potential health benefit of this metabolite, including anti-inflammatory effects and the colonic defence barrier. All of this could be important in our perspective, considering that, to date, NCG/WS patients may be still diagnosed as suffering from IBS. Finally, with regard to NCG/WS, Volta [9] suggests that microbial dysbiosis driven by aberrant changes in the normal composition of the gut microbiota may contribute to intestinal barrier defects and inflammatory responses.

In terms of what induces the dysbiosis, this could be an “erroneous” diet and lifestyle, and/or an epigenetic predisposition. In fact, the first bacterial colonization in utero can modulate immunological and metabolic “fetal programming”, with potential long-term consequences on the risk of developing GI diseases, such as CD and IBD, in addition to allergies, autoimmune and metabolic diseases in the adult life [123,124]. Further, breastfeeding and adult diet are known to strongly influence microbiota. For example, given the scarceness of fiber and the excess of animal fats and proteins, “Western-like diets” are associated with an increase in the secretion of bile salts, resulting in the selection of bile-resistant and sulfate-reducer bacteria with proteolytic and putrefactive action. This is in contrast with a healthy saccharolytic microbiota, promoted, for example, by the “Mediterranean diet”, which allows the preservation of adequate leves of SCFAs and butyrate in particular. Moreover, the richness of Western diets, particularly with regard to trans and satured fats, could result in a greater translocation of LPS and greater inflammation [94,123,124,125,126,127,128,129].

## 4. Implications of the New Hypothesis

Based on the previously described literature and evaluations, it could be considered that NCG/WS is a potentially transient and preventable condition, strongly related to diet quality and balance, rather than to the presence or absence of gluten-containing foods. Without the suggested dysbiotic conditions, the extent of gliadin exposure effects would remain limited to those described in healthy subjects by Hollon [30]. This hypothesis could also be supported by the current lack, to the best of our knowledge, of both a specific and proven genetic background [12,14], and clear and defined evidence of hypersensitivity to gliadin [23,115] in NCG/WS patients.

Regardless, a GFD is currently the cornerstone of treatment for NCG/WS [11]. Undertaking a GFD, if self-administered, means exposure to a series of nutritional risks, and the possibility of obesity and related comorbidities, or in the least excessive weight gain. Moreover, a GFD is usually not economical and is very difficult to follow because of cross-contaminations and/or the presence of small amounts of gluten in food and drugs. In general a GFD appears to be unbalanced and inadequate in terms of both macro- and micronutrients, so it is fundamental that people start a GFD only if, and for the time, strictly necessary [11,50,130,131,132,133,134,135]. Also according to the current NASPGHAN guidelines on GRDs [11], given the uncertainty about the pathogenesis and triggers of NCG/WS, it is not clear if a GFD is actually the optimal treatment for this disease, although it could lead to improved symptoms in self-reported or diagnosed sensitives; it is also not clear how strict and how prolonged this diet should be, and how its efficacy should be monitored independently from the clinical response, given the strong inter-individual differences and the lack of clear guidelines for a standardized follow-up [8,11].

The validation of a co-causal role of specific alterations of the intestinal microbiota in this disorder would be fundamental to the management of NCG/WS patients at a nutritional level. As such, sensitive individuals would no longer need to follow a prolonged, if not permanent, restrictive diet based on the exclusion of gluten. All the more so that this restriction has been shown to affect microbiota richness and composition by reducing beneficial bacteria such as *F. prausnitzii* and *B. longum* [43,136]. On the contrary, by directing the dietetic choice of these patients towards a targeted “prebiotic” type, we could aim to restore a state of eubiosis thanks to the food’s ability to shape the microbiota [126,137,138].

According to what is hypothesized here, targeted probiotic intake could be another fundamental aspect of the treatment of individuals with NCG/WS. In line with this, we might speculate if the weak correlation between magnitude of change in analyzed biomarkers and magnitude of change in symptom score after GFD in the study by Uhde [29] could be explained simply by the absence of an associated probiotic therapy.

One of the chief contributions of our microbiota is its partecipation in food digestion, mainly through a saccharolytic or a proteolytic catabolic pathway [125,139]. With the first one, saccharolytic bacteria, such as *Bifidobacteria* and *Lactobacilli*, hydrolize complex polysaccharides in monomeric sugars, then converted in SCFAs (mainly acetate, propionate, and butyrate). The latter seem to to have a positive role in regulating some physiological processes [94], well summarized in a review by Macfarlane G.T. and Macfarlane S. [139]. For example, SCFAs are known to affect lipid, cholesterol and glucose metabolism in various tissues, and to control the release of satiety hormones; recently, they have also been suggested to play a critical role in the regulation of the gut-microbiota-brain cross talk [94]. Furthermore, SCFAs have been shown to promote intestinal barrier integrity, affect epithelial cell transport and metabolism, epithelial cell growth and differentiation, and elicit direct transcriptional responses in immune cells [125,139]. Both carbohydrate and protein fermentation result in SCFAs production, although in quantitative terms, protein is a minor contributor [139]. Furthermore, besides SCFAs, the proteolytic pathway yields a variety of end-products including co-metabolites such as CO_2_, H_2_, H_2_S, ammonia, amines, thiols, phenols, and indoles, many of which are potentially toxic and are believed to promote the onset of “Western diseases”, such as colon cancer and chronic systemic disorders [125,139]. Saccharolytic bacteria contribute to protect the host from toxic products associated with putrefaction, by requiring them for incorporation into cellular proteins [139]. For these reasons, although the composition of microbiota is a highly personalized peculiarity influenced by several factors such as diet, drugs and lifestyle, it seems that a healthy and balanced microbiota should be mainly saccharolytic with a prevalence of *Bifidobacteria* and *Lactobacilli* [125]. Among SCFAs, butyrate seems to be the most interesting one, thanks to its many important physiological properties [139], some of which have been previously mentioned. Among butyrate-producing bacteria that colonize the human gut, *F. prausnitzii*, *Roseburia* spp., and *E. hallii* belong to the so called “*emerging probiotics*” [100,140]. Of particular interest, *F. prausnitzii* has well characterized immunomodulatory properties in vitro and in vivo [100]: it is able to abolish the NF-kB pathway in intestinal epithelial cells, and prevents colitis in animal models [141]. Currently, *emerging probiotics* culturing and functional characterization difficulties do not allow well-designed pre-clinical and intervention studies yet [140]. For this reason, we have to wait for developing new probiotic products aimed to directly restore butyrate-producing bacteria levels in NCG/WS subjects.

Fortunately, the contribution of cross-feeding interactions between *Bifidobacteria* and butyrate-producing *Firmicutes* is remarkable in the stimulation of butyrate production [99] and thus we believe it could be a strategy in the “attack phase” of NCG/WS treatment. For this purpose, *Bifidobacteria* such as *B. bifidum*, *B. longum* and *B. adolescentis*, the colonic dominant species of the genus, could be useful [97,99]: besides their role in butyrate production, they promote other important anti-inflammatory effects [142,143,144,145,146,147]. For example, some strains of *B. bifidum* have been shown to promote strong anti-inflammatory effects through the inhibition of LPS-induced NF-kB-activation in a strain and dose-dependent manner [142]; some strains of *B. longum* ssp. *longum* and *B. longum* ssp. *infantis*, as well as *B. adolescentis* and *B. animalis* have been shown to induce significant levels of IL-10 in different human cell cultures and in human colonic lamina propria [144]; IL-10 is a regulatory cytokine with an important role in the maintenance of intestinal homeostasis [148,149], and it is absent in gut explants from NCG/WS patients, according to Hollon [30]. *B. longum* ssp. *longum* CCM 7952, is able to increase expression of zonulin-1 and occludin in the intestinal epithelium, preserving TJs and epithelial barrier function [144], thus potentially contributing to a reduction in inflammatory stimuli in the intestinal mucosa of patients with NCG/WS. Finally, *Bifidobacteria*, alone or together with particular *Lactobacilli* and *Streptococci* strains in specific multispecies probiotic mixtures, have been shown to be effective in the prevention and/or treatment of DSS-induced colitis [144,146,150], IBS symptoms [151] and GI inflammatory diseases, such as UC in mice and humans [143,146,147,152,153]. In particular, *Bifidobacterium bifidum* BGN 4 seems to reduce the production of pro-inflammatory cytokines, thanks to the chiro-inositol present in its cell membrane [154]. According to a recent study [155], the administration of a specific probiotic blend containing *Bifidobacterium bifidum* BGN 4, *Lactobacillus salivarius* and *Lactobacillus acidophilus* (Acronelle^®^, Bromatech srl, Milan, Italy) decreases inflammation in patients with IBD, in combination with mesalazine treatment.

Other microbes besides *Bifidobacteria* have demonstrated important anti-inflammatory activities that could be useful in the treatment of NCG/WS: *L. rhamnosus* GG, found in the mucus layer environment [98], has been shown to attenuate intestinal barrier dysfunctions and proinflammatory signals [156], and in particular, to restore in vitro gliadin-induced epithelial barrier disruption and related TEER in the presence of polyamines [157]; *Akkermansia muciniphila*, a Gram-negative strictly anaerobic mucin-degrader, contributes to the production of SCFAs and the maintenance of a healthy protective GI barrier by continuously renovating the mucosae cover [100]*.* The use of probiotic therapy could be strengthened by the association with prebiotics such as resistant starch, inulin, xylans and fructo-oligosaccharides (FOS) which would, directly and indirectly, stimulate butyrate production [94,96,97,98,99,126].

Obviously, if our hypothesis is validated in future studies, further research would be necessary to define the best formulation of treatment, including correct dosages and posology.

## 5. NCG/WS Is a Cancer Risk Factor?

Whether CD is associated with an increased risk of cancer malignancy is still under debate. However, some literature seems to support this hypothesis. A European multi-centre study reported more than a three-fold increased risk of non-Hodgkin’s Lymphoma (NHL) in patients with clinically diagnosed CD [158]. Also an increased incidence and/or mortality for NHL have been reported in patients with CD [159]. An increased risk of GI cancers, duodenal ones in particular, was also documented [160]. On the contrary, other studies suggested that CD may be associated with a reduced risk of some cancers, including breast and lung ones [161,162], although the rationale for this relationship remains unknown.

Overall, it has been observed that the correlation between all types of cancer and CD remained significant for more than one year after diagnosis.

As regards possible correlations between malignancies and NCG/WS, to date data are missing [12]. However, Elfestrom et al. [163] in a study including 28.882 CD patients, 12.860 subjects with small intestinal inflammation (Marsh 1–2) and 3.705 patients with latent CD, found that all the three groups had an increased risk for GI cancers in the first year after diagnosis, but not thereafter. Moreover, an Irish population-based retrospective cohort study [164] reported that malignancy and mortality from malignant neoplasms were increased in patients with positive AGA and negative EmA tests. However, only on the basis of those serological results, the authors could not classify patients as having CD or NCG/WS according to recognised diagnostic criteria, so they could not provide reliable information about a real correlation between NCG/WS and cancer risk.

In conclusion, more investigations are necessary in this field to determine if NCG/WS may be correlated with cancer malignancy.

## 6. Starting Points for Future Research

The data and hypotheses described herein could contribute to the clarification of some controversial aspects of this “young” clinical condition, and could provide some novel avenues for future research. First, it would be interesting to verify the existence of an actual association between the particular dysbiotic profile proposed and NCG/WS. Second, it is of interest to reassess, at the colonic level, the expression of TJPs and TLRs. In this regard, according to a study by Sheth [165] on the effects of LPS on cholangiocytes TJs, LPS disrupts barrier function and increases paracellular permeability in a time- and dose-dependent manner, and also induces a redistribution of TJPs from intercellular junction sites. This study also reported that the LPS-induced disruption of TJs is mediated by TLR4 and LBP [165]. This could raise the question whether, in individuals with NCG/WS, the localization mechanisms of TJPs could be altered, rather than their expression levels. Third, we could speculate that individuals with NCG/WS may have an “overactive” TLR4, attributable to “gain-of-function” mutations. This could also contribute to explain the LPS-induced increase in gut permeability, according to the mechanisms proposed by Guo [57] and Sheth [165], and even more so, may help explain the extent of the immune response caused by the hypothetical combined stimulating effect of ATIs and LPS on the TLR4-MD2-CD14 complex.

Finally, future verification that critical intestinal butyrate levels are pivotal for the onset of NCG/WS could also provide the premise for studies regarding possible associated long-term major complications, such as intestinal lymphoma or gastrointestinal malignancies, as observed in CD. In the current literature there are no such reports, due to a lack of longitudinal data and prospective studies on the natural history of NCG/WS [12]. However, as previously mentioned, butyrate has been suggested to play an important protective role in colorectal carcinogenesis, and has been shown to reduce size and number of tumors in rat models of bowel cancer. Moreover, in vitro, it influences morphology and motility, inhibits proliferation and induces apoptosis in a variety of cancer cells [93,139].

The starting points for future research offered by this paper are described in Table 1.

## 7. Conclusions

This review investigates and discusses, for the first time, novel plausible connections among recent scientific evidence, which have never been linked together in an integrated vision. We also propose a new theory on the pathogenic mechanism of NCG/WS, schematized in Figure 1.

NCG/WS may be considered a multi-factor-onset disorder, potentially transient and preventable, to date without a specific genetic pattern. It may have, instead, an epigenetic component, strongly related to quality and balance of the diet, and consequently, to the microbiota. If the hypotheses posed here are confirmed, NCG/WS could be still defined as a gluten-related disease because of the substantial coexistence of gluten and the stimulating activity of ATIs, for which a GFD is essentially ATIs-free [19].

More precisely, NCG/WS could be considered to be an ATIs/low butyrate-producing *Firmicutes*/low *Bifidobacteria*-dysbiosis-induced disorder, which would more appropriately be referred to as “dysbiosis-induced ATIs sensitivity” (DIAS).

In future, once excluded CD and WA in all their forms, the diagnosis of NCG/WS could ideally be determined also thanks to the aid of the immunological and enterocyte damage biomarkers suggested by Uhde, and the analysis of gut microbiota. According to our hypothesis, host immune system may be mainly addressed towards two different inflammatory stimuli, an exogenous one from the diet (ATIs) and an endogenous one from the resident microbiota, LPS in particular. In our opinion, further studies should be focused into this field, including possible existence of anti-ATIs antibodies. However, first of all, more appropriate diagnostic criteria for NCG/WS and standardized inclusion/exclusion criteria are warranted to perform more reliable studies on it. In our opinion, more appropriate diagnostic criteria are warranted for GRDs in general, because even a certain exclusion of CD still does not seem to be guaranteed, as well as exclusion of non-IgE mediated WA, with a consequent possible contamination of NCG/WS sample, thus creating confounding and possibly biased results. In this regard, it is important to underline that HLA haplotypes should not to be considered as suggestive of NCG/WS: according to the systematic review by Molina-Infante [8], not all studies on this disorder could adequately exclude CD and confirm diagnosis of NCG/WS, as well as not all studies performed, clearly defined and fully described genetic tests, and related the latter to histology [14]. Positive HLA-DQ2/DQ8 haplotypes are necessary, but not sufficient, to develop CD, and their absence excludes the latter, but so far, no genetic markers have been identified for NCG/WS [9], and according to Bardella [14], “other haplotypes, not CD-related, should be investigated”.

According to our hypothesis, the treatment for NCG/WS would be completely different, and no longer necessarily based only on a restrictive and prolonged GFD, but on a targeted “prebiotic” type of nutrition together with specific probiotic therapy, all to be formulated in the future. In particular, we suggest that such a treatment should be specifically addressed to the direct or indirect restoration of adequate levels of butyrate-producing *Firmicutes*, and consequently of intestinal butyrate*.* In fact, in our opinion, the decrease of bp-*F* is at the basis of increased intestinal permeability of NCG/WS, via insufficient butyrate levels, according to the supposed chain reaction. However, we do not know at the moment what should be exactly referred to as “adequate levels” of b-p*F* or intestinal butyrate, also considering that a state of eubiosis results from a balance among all species of our microbiota. Because of culturing and functional characterization difficulties, new strategies are warranted to study the functional group of bp-*F*, later allowing pre-clinical and interventation studies necessary to confirm their usefulness in the treatment of this condition. Furthermore, clinical studies on human are necessary also to define if patients would benefit from an ATI-reduced or -free diet, as well as from IAP and/or butyrate supplementation.

We are aware that the objective which we aim at with this paper could seem ambitious, but we think that it is our duty as part of scientific community, to continuously pose questions and formulate related hypothetical plausible theories, even if not yet supported by experimental evidence: such theories could be at the basis of developing new research projects, which could lead to a step forward in the comprehension of critical aspects in a field, even if negative results are obtained. With this paper, in a very humbly way, we would like to make our idea/intuition available to scientific community, to stimulate it in investigating at various levels about aspects not yet taken into account, and that could have a role in the onset of NCG/WS. In our hypothesis we have critically put together recent scientific evidence in NCG/WS, highlighting diagnostic difficulties and absence of standardized procedures. We are aware that our proposed integrated vision opens up more questions than it closes, by taking into account many aspects which were never been expressly linked together till now in regard to NCG/WS, such as ATIs, IAP, LPS, butyrate and microbiota.

Coordinated studies in different areas of research will be necessary to confirm or reject our hypotheses, and to develop a full understanding of the pathogenesis of NCG/WS, that still seems to retain many intriguing secrets to uncover.

## Figures and Tables

**Table 1:** Starting points for research. The listed starting points are suggested for testing our hypothesis on the pathogenic mechanism of non-coeliac gluten/wheat sensitivity (NCG/WS). b-p*F* = butyrate-producing Firmicutes, IAP = intestinal alkaline phosphatase, TJPs = tight junctions proteins, TLR4 = toll-like receptor 4, ATIs = amylase trypsin inhibitors, LPS = lypopolysaccharide, HDL=high density lipoproteins.

Suggested Starting Points For Testing Our Hypothesis On NCG/WS
1. Association between NCG/WS and dysbiosis, in particular focusing on b-p*F* and *Bifidobacteria* levels
2. Association between NCG/WS and an impaired mucus barrier
3. Roles of butyrate and IAP
4. Presence of TJPs co-localization defects and role in the alteration of gut permeability
5. Expression of TJPs and TLR4 at the colonic level
6. Presence of simultaneous stimulation of the TLR4-MD2-CD14 complex by ATIs and LPS
7. Existence of anti-ATIs antibodies
8. Mutations of TLR4 coding gene and related functional studies
9. Association between NCG/WS and HDL levels
